# Maternal height associated with cesarean section. A cross-sectional study using the 2014–2015 national maternal-child health survey in Guatemala

**DOI:** 10.1186/s12939-020-01182-8

**Published:** 2020-07-31

**Authors:** Evelyn Roldán, Laura M Grajeda, Wilton Pérez

**Affiliations:** Universidad del Valle de Guatemala, 18 Avenida 11-95 Zona 15 Vista Hermosa III, 01015 Guatemala, Guatemala

**Keywords:** Body height, Mothers, Cesarean section, Health surveys, Guatemala

## Abstract

**Background:**

Socioeconomic status is associated with cesarean section (CS). Maternal height, however, may be another related factor to CS. In Guatemala, a quarter of women between 15 and 49 years of age are shorter than 145 cm. Therefore, this study aims to examine the association of maternal height with cesarean section in Guatemala.

**Methods:**

We carried out a secondary analysis study using data from the 2014–15 Guatemalan national maternal and child health survey—9542 mothers aged 15–49 and 12,426 live births were analyzed. We obtained the prevalence ratio of the association between maternal height and CS based on three Poisson regression models. One model included all live births, another the first live birth, and a third model the last live birth. For each model, we accounted for covariates and sampling design.

**Results:**

The national prevalence of CS was 26.3% (95%CI: 25.0, 27.7). The adjusted prevalence ratio of CS, including all live births, was 1.63 (95%CI: 1.37, 1.94) more likely in mothers shorter than 145 cm compared with those equal or greater than 170 cm. This figure was 1.45 (95%CI: 1.19, 1.76) in the model with the first live birth. In the model with the last birth, maternal height was not associated with CS after accounting for previous CS as one of the covariates.

**Conclusions:**

Prevalence of CS in this setting was high and above international recommendations. Further, very short mothers were more likely to experience CS compared to taller mothers after accounting for covariates, except when a previous CS was present. Maternal height should be included in clinical assessments during prenatal care.

## Background

Global health policy focuses on measurable improvements in maternal-child health outcomes. The past millennium development goals 1990–2015, for instance, included maternal mortality as a target. Subsequently, the sustainable development goals (SDG) suggested reducing the mortality rate to less than 70 per 100000 live births by 2015–2030 —implying an average annual decline of at least 7.5% [1]. However, in low- and middle- income countries (LMIC), attaining the SDG 3.1 is a challenge. In Guatemala, for example, the maternal mortality ratio decreased from 205 to 88 per 100000 live births, resulting in an average annual decline of 3.5% during 1990–2015 [1, 2]. Achieving universal and equitable coverage of life-saving interventions is imperative to improve maternal health and meet the SDG 3.1 [2]. In that regard, a properly indicated cesarean section (CS) is a maternal and offspring life-saving intervention implemented in most settings [3]. An ecological study –among 172 WHO member states– found an inverse correlation between country-level prevalence CS in live births and maternal and neonatal mortality [4].

The World Health Organization (WHO) recommends a prevalence of CS between 10 and 15% [5]. Although a low prevalence (< 10%) suggests inadequate access to this surgical procedure, a high prevalence (> 19%) has not shown improvements in perinatal outcomes [4]. A high prevalence of CS may indicate an overuse, and it is associated with adverse health effects for the mother and offspring [6, 7]—especially in settings with poor quality health care facilities [8].

In LMIC, the use of CS is growing and surpassing the WHO recommendation [5]. In a multicountry study, 18.6% of live births were by CS [9]. What is more, the global average rate of CS rose from 6.7 to 19.1% with an annual rate of increase of 4.4% between 1990 and 2014, where the Latin America and the Caribbean region registered highest absolute rate (19.4%) [9].

Previous studies reported medical and socioeconomic factors associated with CS. The medical factors may be determined by maternal, obstetric and fetal categories [8–10]. However, non-medical factors may be associated to CS. For instance, studies have found an association between increasing CS rate and socio-economic status [10, 11] and cultural factors [12, 13]. Another under-studied determinant in LMIC associated with CS is maternal height. Previous research has shown an association between short maternal height and adverse health effects at the time of delivery through labor complications [14–16]. Further, maternal height has been studied as an indicator of economic status and health [17–19]. Shorter women are more likely to experience poverty and social inequalities in access to health care throughout life than taller women [17]. Noteworthily, lower adult height has indicated poor nutritional and social childhood living conditions [20].

A few studies in European and African settings have analyzed the association between maternal height and CS [21, 22]. However, to the best of our knowledge, in Latin America, limited evidence exists on the study of maternal height and cesarean section in a large representative sample. In Guatemala, the mean women’s height is 149.4 cm, the lowest figure worldwide, which contrasts to that reported in countries with taller women (168 cm mean) [23]. Therefore, we conducted secondary data analysis to examine the independent association between CS in live births and maternal height accounted for socioeconomic factors.

## Methods

### Study population and design

**Fig. 1:**
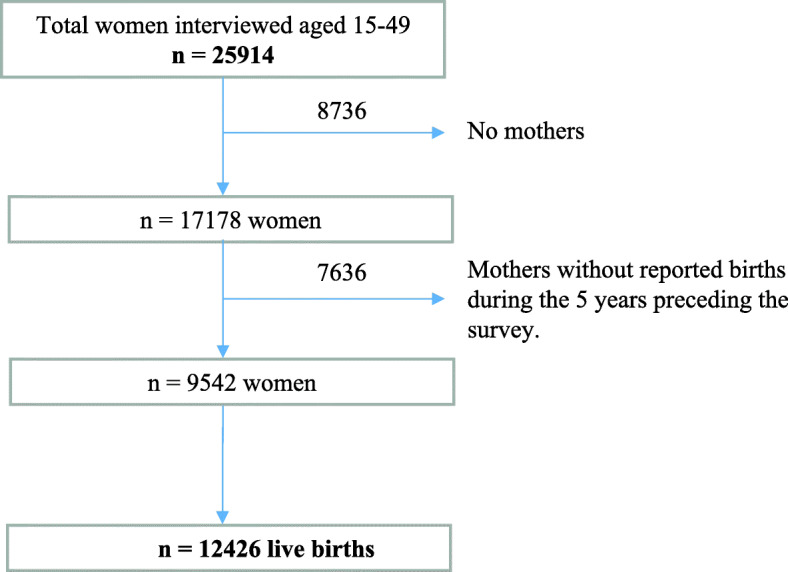
Flow chart of observations used for the analysis, Guatemala 2014–2015

This study is a secondary data analysis based on a cross-sectional design. Data were gathered through the national demographic health survey (DHS) named VI Maternal and Child Survey of Guatemala 2014–2015 (http://www.dhsprogram.com/). The study population were women from 15 to 49 years of age who gave live births in the last 5 years before the survey. The participation rate was 97%. The analytical dataset of this study was constituted of 9542 mothers with complete information on height and type of delivery, which resulted in 12426 live births (Fig. 1).

The sampling procedure is described elsewhere [24]. In brief, the survey used a stratified, two-stage cluster design. In the first stage, clusters were systematically selected with probability proportional to size. Then, in the second stage, households were systematically randomly selected. Each cluster had a mean of 26 households. All women aged 15–49 were interviewed in the selected household.

### Measurements

The 2014–2015 Guatemala DHS measured maternal height in centimeters. To ensure the quality of the body height measurements, trained examiners were standardized using the Habicht method [24, 25]. We adopted the height cut-offs by Arent and colleagues [21]: very short (< 145.0 cm), short (145.0–149.9 cm), short-average (150.0–154.9 cm), average (155.0–159.9 cm), average-tall (160.0–169.9 cm) and tall (≥ 170.0 cm). Type of delivery for each birth was reported by the mother, using the following question: “Was the birth of (name), by cesarean section, that is, did they have to cut your belly to get the baby out? (Yes/No)”. For our analysis, the maternal height and CS were the independent and dependent variables, respectively.

We included covariates such as maternal age in years at childbirth (< 19, 20–29, 30–39, 40+), ethnicity (indigenous, nonindigenous), maternal education (no education, primary, secondary, higher), place of residence (urban, rural), prenatal visits (< 4, 4+), place of birth (public, private, home), skilled birth attendant (yes, no), multiple birth (yes, no), birth order (1, 2–3, 4+), and previous CS (yes, no). The Guatemala DHS computed household wealth index applying principal component analysis (PCA) on all household assets (e.g., TV, radio, mobile) and housing conditions (e.g., type of water, sanitation, wall, floor). Then, the Guatemala DHS categorized the household wealth index in quintiles as poorest, poorer, middle, richer and richest.

### Data analysis

We calculated the prevalence of CS with its respective 95% confidence interval. To assess the association between maternal height and CS, we conducted multivariable Poisson regression models. The Poisson model allowed obtaining the adjusted prevalence ratio, recommended for binary outcomes with a prevalence greater than 10% [26]. Three Poisson models were analyzed. The first model included all live births, the second model included the first live birth and the third model the last live birth. We added a covariate into the regression model when the bivariate association with CS showed a *p*-value < 0.2 in the chi-square test [27]. We assessed the interaction between maternal height and covariates in each model using the “testparm” command. To facilitate interpretation, we dichotomized the maternal height (< 145 cm, ≤ 145 cm) for the interaction analysis. We found significant interaction (*p*-value < 0.05) with ethnicity for the model that included the first live birth. We reported the prevalence ratio, with its respective 95% confidence interval in the models. We did not include the place of birth in the first model as CS was not reported in at home births. A *p*-value < 0.05 was significant. The “svyset” commands were used to account for the complex sampling design. We found no evidence of multicollinearity in all models (VIF < 10). The analysis was done in Stata 14.2 (Stata Corporation, College Station, TX).

### Ethical considerations

This study was a secondary data analysis using public dataset (www.dhsmeasure.com). We obtained consent from The DHS Program to use the dataset. Furthermore, we obtained ethical approval by the Institutional Review Boards at Universidad del Valle in Guatemala with the reference number 188–01-2019. Finally, for this analysis, the dataset is anonymous.

## Results

**Fig. 2:**
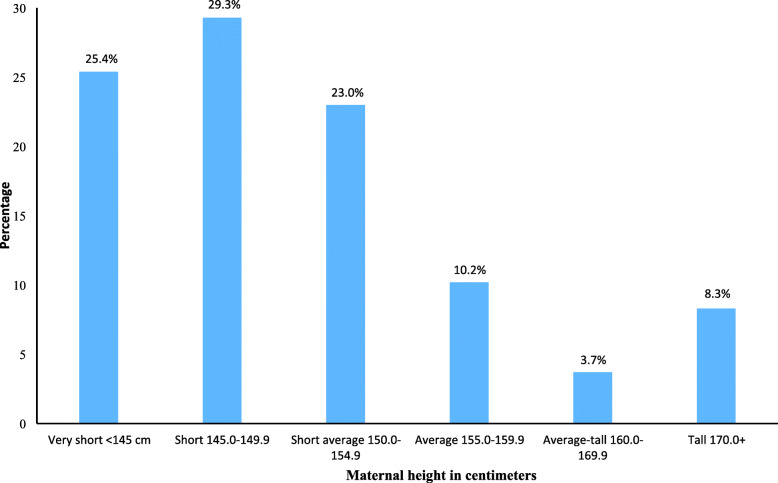
Distribution of women’s height, Guatemala 2014–2018

Figure 2 shows the distribution of maternal height categories. The mean (standard deviation) maternal height was 148.8 cm (6.17), and 25.4% were shorter than 145 cm. Table 1 shows that 58.7% of the deliveries occurred in mothers aged 20–29 and 51.3% in nonindigenous mothers. In half of the births, mothers had only primary education, and half the mothers were in the first (poorest) or second (poorer) wealth index quintile. Almost, two-thirds of live births were in rural areas, and 15.6% were home deliveries. Skilled birth personnel attended 65.4% deliveries, 85% of births had four or more prenatal visits, and 18.3% of mothers reported a previous CS.

**Table 1:** Background characteristics of mothers and bivariate association between covariates and CS, Guatemala 2014–2015

Characteristics	Number of deliveries *n* = 12426	Percentage of deliveries	Prevalence of CS (%)	*p*-value
**Age at birth (years)**				
< 19	1853	14.9	24.2	< 0.001
20–29	7294	58.7	27.9	
30–39	2926	23.6	24.9	
40+	353	2.8	15.7	
**Ethnicity**				
Nonindigenous	6371	51.3	35.5	< 0.001
Indigenous	6051	48.7	17.9	
Missing	4	0.0		
**Maternal education**				
No education	2291	18.4	10.9	< 0.001
Primary	6573	52.9	21.2	
Secondary	3077	24.8	43.0	
Higher	485	3.9	66.7	
**Wealth index quintile**				
Poorest	3394	27.3	10.2	< 0.001
Poorer	2857	23.0	17.4	
Middle	2472	19.9	26.2	
Richer	2142	17.2	42.0	
Richest	1561	12.6	52.7	
**Residence**				
Rural	8158	65.7	19.9	< 0.001
Urban	4268	34.4	37.9	
**Prenatal visits**				
< 4	1861	15.0	27.5	< 0.001
4 or more	10560	85.0	19.6	
Missing	5	0.0		
**Place of birth**				
Public	6187	49.8	34.1	< 0.001
Private	4241	34.1	58.1	
Home	1940	15.6	0	
Missing	58	0.5		
**Skilled birth attendant**				
No	4301	34.6	0	
Yes	8125	65.4	40.3	
**Multiple births**				
No	12209	98.3	25.8	< 0.001
Yes	217	1.8	61.3	
**Birth order**				
1	3972	32.0	36.1	< 0.001
2–3	5011	40.3	28.9	
4+	3443	27.7	11.6	
**Previous cesarean section for the last reported birth*n*** **= 2597**				
No	2121	81.7	7.6	< 0.001
Yes	476	18.3	89.3	

### Maternal and cesarean section association

The national prevalence of CS was 26.3% (95%CI: 25.0, 27.7). Among the first births and last births, the prevalence of CS was 36.1 (95%CI: 34.2, 38.0) and 29.3 (95%CI: 27.9, 30.6) respectively. All covariates were associated with CS with a *p*-value < 0.2, and therefore considered for the modeling (Table 2). Table 2 shows the adjusted prevalence ratio (aPR) for the three models. For model 1 (all live births), the aPR of CS was 1.63 (95%CI: 1.37, 1.94) in very short mothers (< 145 cm) compared to taller (≥ 170 cm). In model 2 (first birth) this figure was 1.45 (95%CI: 1.19, 1.76), and in model 3 for the last birth, this association was not significant after accounting for previous CS which had an aPR of 11.75 (95%CI: 9.93, 13.91). Table 3 shows the interaction analysis in all first live birth. The aPR was significantly higher among shorter mothers (< 145 cm) than taller (≥ 145 cm) in indigenous and nonindigenous. Taller indigenous were less likely to experience cesarean section than taller nonindigenous.

**Table 2:** Unadjusted and adjusted prevalence ratio modeling for the first and last birth, Guatemala 2014–2015

	Adjusted prevalence ratio for all births n = 12417 Model 1^a^	95% Confidence Interval	Adjusted prevalence ratio for first birth *n* = 3160 Model 2^b^	95% Confidence Interval	Adjusted prevalence ratio for last birth *n* = 1448 Model 3^c^	95% Confidence Interval
Maternal height cm						
Very short < 145 cm	1.63	(1.37 ─ 1.94)	1.45	(1.19 ─ 1.76)	1.31	(0.90 ─ 1.92)
Short 145.0–149.9	1.36	(1.14 ─ 1.62)	1.14	(0.94 ─ 1.39)	1.16	(0.79 ─ 1.70)
Short average 150.0–154.9	1.28	(0.07 ─ 1.52)	1.05	(0.86 ─ 1.28)	1.19	(0.82 ─ 1.72)
Average 155.0–159.9	1.40	(1.17 ─ 1.66)	1.12	(0.90 ─ 1.38)	1.38	(0.93 ─ 2.04)
Average-tall 160.0–169.9	1.27	(1.02 ─ 1.56)	0.93	(0.72 ─ 1.21)	1.24	(0.81 ─ 1.88)
Tall ≥170.0	1.00	Reference	1.00	Reference	1.00	Reference

^a^ Adjusted for: age at birth, ethnicity, education, wealth index, residence, prenatal visits, multiple births and birth order

^b^Adjusted for: age at birth, ethnicity, education, wealth index, residence, prenatal visits, place of birth and skilled birth attendant

^c^Adjusted for: age at birth, ethnicity, education, wealth index, residence, prenatal visits, place of birth, skilled birth attendant and previous cesarean section

**Table 3:** Cesarean section prevalence ratio in relation to ethnicity and maternal height for the first live birth, Guatemala 2014–2015

Ethnicity	Maternal height cm	Adjusted prevalence ratio^a^	95% Confidence interval	*p*-value
Nonindigenous	< 145	1.19	(1.02 ─ 1.38)	< 0.01
	≥ 145	1.00	Reference	
Indigenous	< 145	1.29	(1.14 ─ 1.47)	< 0.05
	≥ 145	0.87	(0.77 ─ 0.98)	

^a^Adjusted for: age at birth, education, wealth index, residence, prenatal visits, place of birth and skilled birth attendant

In both model 1 and model 2, mothers over 30 were more likely to have a CS compared to the other younger age groups of mothers. However, in the model with the last birth, CS was significantly higher in mothers aged 30–39 compared to mothers under 19. By socioeconomic status, CS was most likely in mothers in the richest quintile compared to those in the poorest quintile. Regarding ethnicity, indigenous mothers had less chance than nonindigenous of CS, although, in model 2, it was not significant. In the model of all live births, multiple births had 2.7 more chance of CS than single births. Those births of higher-order were more likely to experience a CS compared to a birth of first order. (Additional File 1).

## Discussion

This large representative national study included live birth deliveries from mothers of reproductive age, examined the frequency of cesarean sections and its relationship with maternal height. We found a prevalence of cesarean section above the WHO recommendation. Lower maternal height independently associated to cesarean section, except when the model accounted for previous cesarean delivery.

Our findings show that the prevalence of CS is increasing in Guatemala. In 2002, the national prevalence of CS was 11.2%, less than half of the reported in this study (26.3%) [24]. This finding is in line with previous studies from low-income settings where vaginal delivery is becoming a less utilized mode of delivery and CS is becoming more common [28]. Notably, the evidence has shown no further health benefits for the mother and offspring with a prevalence of CS above 19%, and thus, overuse of this surgery may be occurring [4]. A Guatemalan study carried out from 2010 to 2016 included more 30000 deliveries and reported that out of the 18% CS only 10% were for life-saving indications [29].

The association between maternal height and CS analyzing all live births and the first birth was consistent with the findings of a study in sub-Saharan Africa settings, where mothers whose height was less than 145 cm had twice the probability of CS compared to mothers with a height of 145 cm or above [21]. An explanation is that maternal height may represent an obstetric risk during delivery. Mothers of very short (< 145 cm) stature have elevated risk of labor obstruction attributable to cephalopelvic disproportion [14–16, 30].

Another potential factor that contributes to the association between short maternal stature and cesarean is the nutritional status. In Brazil, shorter women were more likely to be overweight or obese than those taller [19], and excess weight has been found to increase CS [31]. Furthermore, a systematic review showed increased odds of newborns with large size for gestational age, higher birth weight and macrosomia in offspring whose mothers were overweight/obese before pregnancy than in those whose mothers’ weight was normal [32]. Thus, overweight and obesity previous to or during pregnancy in short/very short mothers may prolong labor and therefore indicate a CS [33]. The former has public health implications in Guatemala as overweight and obesity in 2014 affected 85% of women aged 15–49 [34] and half of the non-pregnant women with the intention to conceive [35].

Maternal height has been studied as a socioeconomic indicator for social and health inequalities [20]. Short mothers are more likely to experience barriers to access to high-quality health care services [36]. In our study sample, the percentage of very short mothers (< 145 cm) was higher in rural, primary/non-educated, poorer/poorest and indigenous compared to their counterparts (Additional file 2). The lower socioeconomic position of women reduces access to health and reproductive health-seeking behavior [36, 37]. Therefore, mothers with short/very short height may have experienced socioeconomic, physical and cultural barriers to access to delivery facilities. The delay for seeking healthcare may motivate medical staff to recommend a CS to prevent adverse maternal and offspring outcomes during labor [37]. However, further studies are needed to document the extent of the association between healthcare-seeking delay for delivery and CS.

For the last birth model, after accounting for previous CS, the association between maternal height and CS was not significant. In our study, 68.4% of CS (data not shown) reported in the most recent singleton birth were performed on mothers who had had a previous cesarean delivery. The lack of association reported in this study was not a surprise, as, in many settings, CS is indicated when the mother has had a previous CS [38, 39].

We found 15.6% of births were home deliveries. Among these deliveries, 36.9% were from very short mothers, as compared to 10.6% in taller mothers. Further, a higher proportion of mothers were indigenous (75.0%) in this group of home deliveries. According to a 2013 survey by Salud Mesoamerica Initiative (SMI), Guatemalan indigenous women were less likely to have an institutional delivery compared to nonindigenous [40]. SMI also found that partial and complete prenatal care was associated with higher odds of in-facility delivery compared to those who did not receive any prenatal care [41]. We found that complete prenatal care (4 or more visits) was higher in taller mothers compared to very short—although it was not significant (Additional file 2). Therefore, further research is needed to analyze the social circumstances experienced by very short mothers delivering at home and the health maternal and offspring outcomes.

### Strengths and limitations

This study has strengths and limitations. One strength is the large sample with national representation. The participation rate was 97%, which reduces the potential for selection bias. The proportion of missing information was very low (< 0.5%).

There is a potential to recall bias in terms of self-reporting of CS as reported elsewhere [42]. We try to overcome this issue by constraining our analysis using the first and last birth. There are several variables related to CS not collected during the survey due to the cross-sectional design. For instance, gestational age at the time of delivery, information regarding whether the CS was an elective or emergency procedure, maternal nutrition during pregnancy (e.g., obesity), onset of labor, cephalic circumference and fetal presentation. As we used the data from a cross-sectional survey, we only reported the association between maternal height and CS without assuming a causal relationship.

## Conclusions

In Guatemala, the prevalence of CS deliveries is high and above international recommendations. Besides, CS was used more frequently in very short mothers. However, this association lost significance when previous CS was accounted for. One implication from our findings is that the health system needs to explore the reasons for this association and the social circumstances experienced by the mother.

Furthermore, our study findings reinforce -indirectly- the importance of implementing comprehensive and cost-effective health and nutrition intervention policies as short adult height represents the accumulated effect of social, health and nutrition deprivations across generations [43–46].

## Supplementary information

**Additional file 1.** Adjusted prevalence ratio for the three models.

**Additional file 2.** Live birth distribution by maternal height and social and obstetric characteristics.

## Data Availability

Requests for the data must be made to The DHS Program at https://dhsprogram.com.

## Abbreviations

95% CI: 95% confidence interval
CS: Cesarean Section
DHS: Demographic Health Survey
LMIC: Low- and middle-income countries
SDG: Sustainable Development Goals
SMI: Salud Mesoamerica Initiative
VIF: Variance inflation factor
WHO: World Health Organization
