# Supplementary material for: Maternal height associated with cesarean section. A cross-sectional study using the 2014–2015 national maternal-child health survey in Guatemala
Source: Int J Equity Health. 2020 Jul 31;19:95. doi: 10.1186/s12939-020-01182-8 (PMC7393904; doi:10.1186/s12939-020-01182-8)
Supplement: Supplementary file 1 — Additional file 1. Adjusted prevalence ratio for the three models. [file 12939_2020_1182_MOESM1_ESM.docx]

Additional File 1. Adjusted prevalence ratio for the three models.

|  | Adjusted prevalence ratio for all births  n = 12417  Model 1^1^ | 95% Confidence Interval | Adjusted prevalence ratio for first birth  n = 3160  Model 2^2^ | 95% Confidence Interval | Adjusted prevalence ratio for last birth  n = 1448  Model 3^3^ | 95% Confidence Interval |
| --- | --- | --- | --- | --- | --- | --- |
| **Age at birth (years)** |  |  |  |  |  |  |
| <19 | 1.00 | Reference | 1.00 | Reference | 1.00 | Reference |
| 20-29 | 1.26 | (1.13 ─ 1.42) | 1.22 | (1.08 ─ 1.37) | 1.14 | (0.93 ─ 1.41) |
| 30-39 | 1.70 | (1.46 ─ 1.98) | 1.41 | (1.16 ─ 1.70) | 1.33 | (1.06 ─ 1.67) |
| 40+ | 1.66 | (1.25 ─ 2.19) | 1.70 | (1.17 ─ 2.46) | 1.59 | (0.90 ─ 2.81) |
| **Ethnicity** |  |  |  |  |  |  |
| Nonindigenous | 1.00 | Reference | 1.00 | Reference | 1.00 | Reference |
| Indigenous | 0.72 | (0.66 ─ 0.79) | 0.91 | (0.83 ─ 1.00) | 0.91 | (1.80 ─ 1.02) |
| **Maternal education** |  |  |  |  |  |  |
| No education | 1.00 | Reference | 1.00 | Reference | 1.00 | Reference |
| Primary | 1.31 | (1.09 ─ 1.57) | 0.97 | (0.77 ─ 1.22) | 1.13 | (0.87 ─ 1.48) |
| Secondary | 1.56 | (1.28 ─ 1.89) | 0.96 | (0.77 ─ 1.21) | 1.05 | (0.80 ─ 1.37) |
| Higher | 1.87 | (1.50 ─ 2.32) | 1.14 | (0.89 ─ 1.46) | 1.18 | (0.84 ─ 1.65) |
| **Wealth index quintile** |  |  |  |  |  |  |
| Poorest | 1.00 | Reference | 1.00 | Reference | 1.00 | Reference |
| Poorer | 1.47 | (1.26 ─ 1.73) | 1.15 | (0.94 ─ 1.40) | 1.01 | (0.78 ─ 1.31) |
| Middle | 1.90 | (1.63 ─ 2.20) | 1.25 | (1.03 ─ 1.51) | 1.09 | (0.87 ─ 1.37) |
| Richer | 2.56 | (2.19 ─ 2.99) | 1.45 | (1.19 ─ 1.78) | 1.16 | (0.92 ─ 1.46) |
| Richest | 2.66 | (2.26 ─ 3.12) | 1.44 | (1.16 ─ 1.79) | 1.10 | (0.82 ─ 1.47) |
| **Residence** |  |  |  |  |  |  |
| Rural | 1.00 | Reference | 1.00 | Reference | 1.00 | Reference |
| Urban | 1.03 | (0.95 ─ 1.12) | 1.02 | (0.91 ─ 1.14) | 1.03 | (0.91 ─ 1.16) |
| **Prenatal visit** |  |  |  |  |  |  |
| < 4 | 0.95 | (0.83 ─ 1.09) | 1.11 | (0.95 ─ 1.30) | 0.91 | (0.80 ─ 1.05) |
| 4 or more | 1.00 | Reference | 1.00 | Reference | 1.00 | Reference |
| **Place of birth** |  |  |  |  |  |  |
| Public |  |  | 1.00 | Reference | 1.00 | Reference |
| Private |  |  | 1.37 | (1.22 ─ 1.55) | 1.09 | (0.94 ─ 1.27) |
| **Skilled birth attendant** |  |  |  |  |  |  |
| No |  |  | 1.00 | Reference | 1.00 | Reference |
| Yes |  |  | 2.51 | (0.98 ─ 6.43) | 2.15 | (0.73 ─ 6.32) |
| **Multiple births** |  |  |  |  |  |  |
| No | 1.00 | Reference |  |  |  |  |
| Yes | 2.70 | (2.28 ─ 3.20) |  |  |  |  |
| **Birth order** |  |  |  |  |  |  |
| 1 | 1.00 | Reference |  |  |  |  |
| 2-3 | 0.75 | (0.69 ─ 0.80) |  |  |  |  |
| 4+ | 0.36 | (0.31 ─ 0.41) |  |  |  |  |
| **Previous cesarean section** |  |  |  |  |  |  |
| No |  |  |  |  | 1.00 | Reference |
| Yes |  |  |  |  | 5.50 | (4.67 ─ 6.48) |
