# Supplementary material for: Maternal height associated with cesarean section. A cross-sectional study using the 2014–2015 national maternal-child health survey in Guatemala
Source: Int J Equity Health. 2020 Jul 31;19:95. doi: 10.1186/s12939-020-01182-8 (PMC7393904; doi:10.1186/s12939-020-01182-8)
Supplement: Supplementary file 2 — Additional file 2. Live birth distribution by maternal height and social and obstetric characteristics. [file 12939_2020_1182_MOESM2_ESM.docx]

Additional File 2. Live birth distribution by maternal height and social and obstetric characteristics

|  | Very short <145 cm  n = 3269 | | Short 145.0-149.9  n = 3658 | | Short average 150.0-154.9  n = 2769 | | Average 155.0-159.9  n = 1299 | | Average-tall 160.0-169.9  n = 425 | | Tall ≥170.0  n = 1006 | | p-value |
| --- | --- | --- | --- | --- | --- | --- | --- | --- | --- | --- | --- | --- | --- |
| Characteristics | n | Percentage | n | Percentage | n | Percentage | n | Percentage | n | Percentage | n | Percentage |  |
| **Age at birth (years)** |  |  |  |  |  |  |  |  |  |  |  |  |  |
| <19 | 456 | 13.95 | 563 | 15.39 | 416 | 15.02 | 182 | 14.01 | 60 | 14.12 | 176 | 17.50 | p < 0.01 |
| 20-29 | 1766 | 54.02 | 2128 | 58.17 | 1657 | 59.84 | 807 | 62.12 | 271 | 63.76 | 665 | 66.10 |  |
| 30-39 | 908 | 27.78 | 864 | 23.62 | 628 | 22.68 | 282 | 21.71 | 88 | 20.71 | 156 | 15.51 |  |
| 40+ | 139 | 4.25 | 103 | 2.82 | 68 | 2.46 | 28 | 2.16 | 6 | 1.41 | 9 | 0.89 |  |
| **Ethnicity** |  |  |  |  |  |  |  |  |  |  |  |  |  |
| Nonindigenous | 1078 | 33.02 | 1721 | 47.05 | 1734 | 62.62 | 1001 | 77.06 | 360 | 84.71 | 477 | 47.42 | p < 0.01 |
| Indigenous | 2187 | 66.98 | 1937 | 52.95 | 1035 | 37.38 | 298 | 22.94 | 65 | 15.29 | 529 | 52.58 |  |
| **Maternal education** |  |  |  |  |  |  |  |  |  |  |  |  |  |
| No education | 962 | 29.43 | 690 | 18.86 | 310 | 11.20 | 95 | 7.31 | 26 | 6.12 | 208 | 20.68 | p < 0.01 |
| Primary | 1823 | 55.77 | 2101 | 57.44 | 1401 | 50.60 | 544 | 41.88 | 126 | 29.65 | 578 | 57.46 |  |
| Secondary | 443 | 13.55 | 779 | 21.30 | 911 | 32.90 | 542 | 41.72 | 216 | 50.82 | 186 | 18.49 |  |
| Higher | 41 | 1.25 | 88 | 2.41 | 147 | 5.31 | 118 | 9.08 | 57 | 13.41 | 34 | 3.38 |  |
| **Wealth index quintile** |  |  |  |  |  |  |  |  |  |  |  |  |  |
| Poorest | 1290 | 39.46 | 1067 | 29.17 | 515 | 18.60 | 152 | 11.70 | 16 | 3.76 | 3.54 | 35.19 | p < 0.01 |
| Poorer | 882 | 26.98 | 917 | 25.07 | 547 | 19.75 | 194 | 14.93 | 51 | 12.00 | 266 | 26.44 |  |
| Middle | 617 | 18.87 | 722 | 19.74 | 610 | 22.03 | 290 | 22.32 | 68 | 16.00 | 165 | 16.40 |  |
| Richer | 344 | 10.52 | 644 | 17.61 | 605 | 21.85 | 301 | 23.17 | 130 | 30.59 | 118 | 11.73 |  |
| Richest | 136 | 4.16 | 308 | 8.42 | 492 | 17.77 | 362 | 27.87 | 160 | 37.65 | 103 | 10.24 |  |
| **Residence** |  |  |  |  |  |  |  |  |  |  |  |  |  |
| Rural | 2375 | 72.65 | 2489 | 68.04 | 1670 | 60.31 | 719 | 55.35 | 195 | 45.88 | 710 | 78.58 | p < 0.01 |
| Urban | 894 | 27.35 | 1169 | 31.96 | 1099 | 39.69 | 580 | 44.65 | 230 | 54.12 | 296 | 29.42 |  |
| **Prenatal visit** |  |  |  |  |  |  |  |  |  |  |  |  |  |
| < 4 | 554 | 16.96 | 552 | 15.09 | 405 | 14.63 | 166 | 12.78 | 33 | 7.76 | 151 | 15.01 | p = 0.0666 |
| 4 or more | 2712 | 83.04 | 3105 | 84.91 | 2363 | 85.37 | 1133 | 87.22 | 3.92 | 92.24 | 855 | 84.99 |  |
| **Place of birth** |  |  |  |  |  |  |  |  |  |  |  |  |  |
| Public | 1519 | 46.47 | 1925 | 52.62 | 1457 | 52.62 | 666 | 51.35 | 214 | 50.35 | 406 | 40.36 | p < 0.01 |
| Private | 210 | 6.42 | 417 | 11.40 | 583 | 21.05 | 430 | 33.15 | 184 | 43.29 | 116 | 11.53 |  |
| Home | 1531 | 46.83 | 1302 | 35.59 | 713 | 25.75 | 194 | 14.96 | 24 | 5.65 | 477 | 47.42 |  |
| Missing | 9 | 0.28 | 14 | 0.38 | 16 | 0.58 | 7 | 0.54 | 3 | 0.71 | 7 | 0.70 |  |
| **Skilled birth attendant** |  |  |  |  |  |  |  |  |  |  |  |  |  |
| No | 1545 | 47.26 | 1323 | 36.17 | 723 | 26.11 | 193 | 14.86 | 31 | 7.29 | 486 | 48.31 | p < 0.01 |
| Yes | 1724 | 52.74 | 2335 | 63.83 | 2046 | 73.89 | 1106 | 85.14 | 3.94 | 32.71 | 520 | 51.69 |  |
| **Multiple births** |  |  |  |  |  |  |  |  |  |  |  |  |  |
| No | 3198 | 97.83 | 3594 | 98.25 | 2727 | 98.48 | 1275 | 98.15 | 415 | 97.65 | 1000 | 99.40 | p < 0.01 |
| Yes | 71 | 2.17 | 64 | 1.65 | 42 | 1.52 | 24 | 1.85 | 10 | 2.35 | 6 | 0.60 |  |
| **Birth order** |  |  |  |  |  |  |  |  |  |  |  |  |  |
| 1 | 875 | 26.77 | 1113 | 30.43 | 933 | 33.69 | 496 | 38.18 | 189 | 44.47 | 366 | 36.38 | p < 0.01 |
| 2-3 | 1180 | 36.10 | 1479 | 40.43 | 1198 | 43.26 | 576 | 44.34 | 188 | 44.24 | 390 | 38.77 |  |
| 4+ | 1214 | 37.14 | 1066 | 29.14 | 6.38 | 23.04 | 227 | 17.47 | 48 | 11.29 | 250 | 24.88 |  |
| **Previous cesarean section** |  |  |  |  |  |  |  |  |  |  |  |  |  |
| No | 679 | 84.98 | 679 | 83.21 | 391 | 77.43 | 167 | 70.76 | 33 | 62.26 | 172 | 91.49 | p < 0.01 |
| Yes | 120 | 15.02 | 137 | 16.79 | 114 | 22.57 | 69 | 29.24 | 20 | 37.74 | 16 | 8.51 |  |
